# Sustained Delivery of Chondroitinase ABC from Hydrogel System

**DOI:** 10.3390/jfb3010199

**Published:** 2012-03-19

**Authors:** Filippo Rossi, Pietro Veglianese, Marco Santoro, Simonetta Papa, Cristina Rogora, Valentina Dell’Oro, Gianluigi Forloni, Maurizio Masi, Giuseppe Perale

**Affiliations:** 1 Department of Chemistry, Materials and Chemical Engineering “Giulio Natta”, Politecnico di Milano, via Mancinelli 7, Milan 20131, Italy; E-Mails: marco.santoro@chem.polimi.it (M.S.); maurizio.masi@polimi.it (M.M.); giuseppe.perale@polimi.it (G.P.); 2 Department of Neuroscience, Mario Negri Institute for Pharmacological Research, via La Masa 19, Milan 20156, Italy; E-Mails: pietro.veglianese@marionegri.it (P.V.); simonetta.papa@marionegri.it (S.P.); cristina.rogora@marionegri.it (C.R.); valentina.delloro@marionegri.it (V.D.); gianluigi.forloni@marionegri.it (G.F.)

**Keywords:** chondroitinase ABC, hydrogels, drug delivery, spinal cord injury

## Abstract

In the injured spinal cord, chondroitin sulfate proteoglycans (CSPGs) are the principal responsible of axon growth inhibition and they contribute to regenerative failure, promoting glial scar formation. Chondroitinase ABC (chABC) is known for being able to digest proteoglycans, thus degrading glial scar and favoring axonal regrowth. However, its classic administration is invasive, infection-prone and clinically problematic. An agarose-carbomer (AC1) hydrogel, already used in SCI repair strategies, was here investigated as a delivery system capable of an effective chABC administration: the material ability to include chABC within its pores and the possibility to be injected into the target tissue were firstly proved. Subsequently, release kinetic and the maintenance of enzymatic activity were positively assessed: AC1 hydrogel was thus confirmed to be a feasible tool for chABC delivery and a promising device for spinal cord injury topic repair strategies.

## 1. Introduction

The well known consequences of traumatic spinal cord injury (SCI), e.g*.*, dramatically reduced quality of life in patients and burdensome impact on the public economy, make of it one of the most challenging neurological conditions to investigate [1,2,3]. SCI is the result of a primary event due to contusive, compressive or stretch injury, which is followed by the so-called “secondary injury”: the latter is considered the main cause of post traumatic neural degeneration of the cord [4,5]. It is now well understood that the primary target for neuroprotection is to counteract the mechanisms of this secondary injury in order to minimize its pathological consequences [6]. The initial insult is followed by numerous barriers to successful axon regeneration, including glial scarring that produces chemical signals inhibiting axonal recovery via chondroitin sulfate proteoglycan (CSPGs) upregulation [7].

*In vivo* studies provided key evidences that chondroitinase ABC (chABC), a bacterial enzyme able to digest chondroitin sulfate glycosaminoglycans (CS-GAGs) of CSPGs, fosters axonal regeneration and sprouting, promotes plasticity of uninjured pathways and, most relevantly, supports functional recovery in various animal models [5,7,8,9]. However, chABC delivery is severely limited due to its deactivation and biodistribution, as classical intrathecal deliveries require repeated administrations and are thus exposed to recurrent infections [5,7]. These drawbacks can be overcome by using smart drug delivery systems, providing local sustained release able to both reduce systemic side effects of drugs and increase treatment efficacy [10,11]. Among these systems, hydrogels seem to be very suitable for SCI therapies due to their characteristic properties: (I) the ability to retain water, thus mimicking living tissues; (II) high biocompatibility; III) the possibility to allow precisely controlled release rates [12,13]. 

In this study an injectable hydrogel from agarose and Carbomer 974P, here briefly named agarose-carbomer (AC) in accordance with previous works [14,15,16], was used as a potential platform for chABC controlled delivery system. In our previous studies, biocompatibility of this hydrogel-based system was positively assessed and its feasibility as multiple drug delivery tool in SCI repair strategies was proven [17]. Multiple release profiles were achieved by loading gel with a combination of molecules with high and low hydrodynamic radii that mimic neuroprotective and neuroregenerative molecules respectively.

*In vitro*, *in vivo* and *ex vivo* release studies showed an independent combination of fast diffusion-controlled kinetics for smaller molecules together with slow diffusion-controlled kinetics for larger ones. Moreover, a preserved functionality of loaded substances was always achieved, confirming the absence of any chemically stable interactions between gel matrix and loaded molecules.

Here, release tests were first performed with Texas-Red dextran (Tx) to confirm the possibility of achieving an effectively deliver of chABC-like molecules [18,19]. Delivery tests on chABC-loaded hydrogel were then performed in order to investigate the capability of preserving chABC enzymatic activity also after delivery. 

A key quality for a delivery system is to be neutral, *i.e.*, being capable of maintaining the activity of the carried substance: chABC activity was here specifically assessed through its efficacy of digesting decorin, a small proteoglycan [7].

## 2. Experimental Section

### 2.1. Hydrogel Synthesis

Carbomer 974P (a highly branched polyacrylic acid) was provided by Fagron (Rotterdam, The Netherlands), high purity sodium hydroxide (NaOH) was purchased by Fluka (Buchs, Switzerland), and agarose was provided by Invitrogen (Carlsbad, CA, USA). The solvent used was PBS (Phosphate Buffer Saline), purchased by Sigma-Aldrich (Taufkirchen, Germany).

Hydrogels were synthesized by bulk reaction in PBS between agarose and Carbomer (here briefly called *AC1* hydrogel in according with previous studies [14,15,16,20]). Polymeric solution was constituted by 1 g of agarose and 0.5 g carbomer solvated in 100 mL of PBS, adding NaOH 1N to keep a neutral pH. The onset of gelation was achieved by means of electromagnetic (EM) stimulation (500 W irradiated power) heating in ratio of 1 min per 10 mL of polymeric solution at 80 °C. 

### 2.2. Morphological Studies: Environmental Scanning Electron Microscopy (E/SEM) Analysis

Environmental scanning electron microscopy analysis was performed on gold sputtered samples at 10 kV with Evo 50 EP Instrumentation (Zeiss, Jena, Germany). To preserve the actual morphology of the hydrogel under complete swelling, freeze-drying (24 h) was applied to remove all the liquid phase by sublimation. Because of the low operating values of temperature and pressure of this procedure, gel polymer chains retain the same conformation they show in wet conditions [21,22].

### 2.3. Rheology

The storage moduli and time sweep of *AC1* hydrogel were performed at 25 °C using a Rheometric Scientific ARES instrumentation (TA Instruments, New Castle, DE, USA) equipped with 30 mm parallel plates, with a 4 mm gap between them [15]. Oscillatory responses (G', elastic modulus, and G'', loss/viscous modulus) were determined at low values of strain (0.02%) over the frequency range 0.1–100 rad/s. A dynamic stress sweep test was conducted on the material to determine its linear viscoelastic region (LVR). During a stress sweep test, the sample is subjected to an increasing stress, while frequency and temperature are maintained constant.

### 2.4. Tx Loading and Release Studies

Tx, being of about 70 kDa molecular weight (Invitrogen, Eugene, OR, USA), was chosen because of its hydrodynamic radius similar to chABC [18,19] and for its easy detectability by UV spectroscopy. Tx is not temperature sensitive so, loading before gelation is possible: a Tx water solution (1 mg/mL) was added to the polymeric formulate while still at sol stage and then the final mixture was left to gel, as described above. At the end of gelation process Tx molecules remain solvated into water, entrapped within hydrogel pores [15,16]. Samples were then placed in a well filled with distilled water at 25 °C, several aliquots were withdrawn at different time points, and analyzed by UV spectroscopy to calculate Tx mass release [15,16]. 

### 2.5 Delivery and Enzymatic Activity Evaluation

25 μL chABC (Seikagaku Biobusiness Corp., Tokyo, Japan), with a concentration of 2 U/0.5 mL in PBS, was mixed with 25 μL of hydrogel, once gel had already been irradiated and cooled down to 37 °C. chABC remained homogeneously entrapped in the pores of the hydrogel at the end of gelation process, similarly to what happened inside Tx-loaded hydrogels. Every well containing a hydrogel was filled with 100 μL of distilled water and stored at room temperature. Aliquots of 100 μL were collected from every gel samples at defined time points (1, 2, 5 and 7 days), filling wells with fresh solution. Subsequently, 10 μL of decorin 2 mg/mL (Sigma-Aldrich, Taufkirchen, Germany) was added to 40μL of each samples and enzymatic digestion was allowed to proceed for 12 hours at 37 °C. The resulting products were analyzed by sodium dodecyl sulphate-polyacrylamide gel electrophoresis (SDS-PAGE), in order to assess any potential adverse effects of the hydrogel on chABC enzymatic activity. Solutions of pure decorin and decorin digested by chABC were used as controls during SDS-PAGE analysis.

### 2.6. Statistical Analysis

Where applicable, experimental data were analyzed using Analysis of Variance (ANOVA). Statistical significance was set to p value <0.05. Results are presented as mean value ± standard deviation [23].

## 3. Results and Discussion

**Figure 1:**
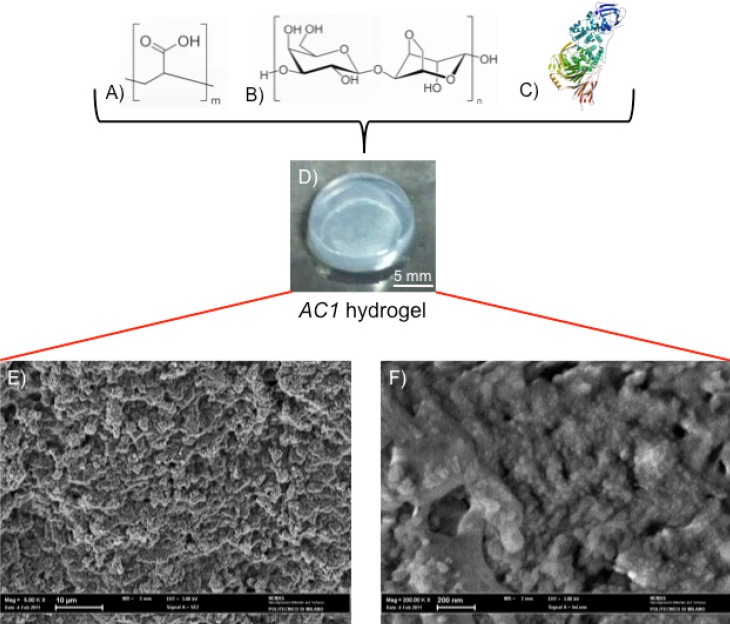
Carbomer 974P (**A**); agarose (**B**) were chemically cross-linked to form hydrogel in phosphate buffer saline solution. Esterification, hydrogen bonding and carboxylation bring polymer chains statistically closer, thus creating a stable heterogeneous structure. The gelling solution was homogenized together with chondroitinase ABC (chABC); (**C**)above sol-gel transition temperature. From a macroscopic point of view the resulting material appears as in (**D**) (scale bar = 5 mm). From a microscopic point of view the hydrogel is densely structured, as observable by ESEM analysis [(**E**) scale bar = 10 μm; (**F**) scale bar = 200 nm].

The investigated hydrogel is a chemical gel synthesized through a statistical block polycondensation between Carbomer 974P (Figure 1A) and agarose (Figure 1B) [15,16]. Before the sol/gel transition takes place, chABC (Figure 1C) is loaded and it thus results physically entrapped within the three-dimensional polymeric network of the gel. From a macroscopic point of view, the resulting material appears as in Figure 1D; the highly entangled nanostructure is confirmed by ESEM analysis (as depicted in Figure 1E,F).

**Figure 2:**
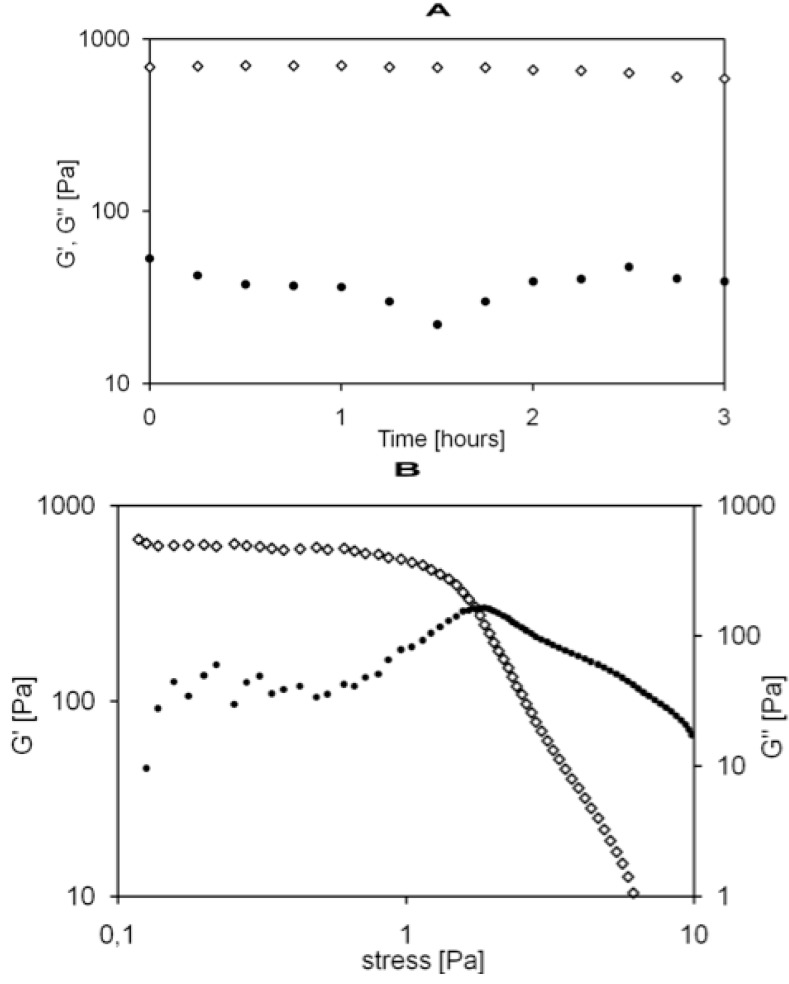
(**A**) Mechanical spectra of agarose-carbomer (AC1) gel with small oscillatory shear in the linear viscoelastic regime: **◇**G', **•**G'' expressed in Pa *vs*. time in hours; (**B**) dynamic strain sweep experiments of AC1 gel (**◇**G', **•**G'') in Pa *vs*. stress in Pa.

Indeed, Carbomer 974 P carboxylic groups constitute the main cross-linking sites to be reacted with hydroxyl groups from agarose, altogether giving rise to the three dimensional matrix. These polymers were selected because of their well-known biocompatibility in central nervous system (CNS). Agarose has been widely used in biomaterials for spinal cord injury repair strategies [24], while carbomer was chosen because of its anti-inflammatory properties and proved biocompatibility [25]. The resulting hydrogel holds great promise, due to the several positive aspects demonstrated in previous studies, such as: biocompatibility, high structural versatility, thixotropic nature and capability of being a controlled delivery tool [14,16]. At first, the synthesized material was studied in terms of mechanical properties. Figure 2A shows the Dynamic Frequency Sweep test (DFS) spectra performed at 25 °C as a function of time: storage modulus (G') is approximately one order of magnitude higher than the loss modulus (G''), exhibiting an elastic behavior rather than a viscous one. Moreover, G' and G'' are independent from time and frequency [26], matching the characteristic signature of a solid-like material.

The G' and G'' moduli trend versus oscillation stress is reported in Figure 2B. Within the linear viscoelastic region (LVR), G' values were always greater than the G'' ones, demonstrating AC1 hydrogel remarkable elasticity. G' and G'' do not change within the range of the LVR, suggesting that the structure of the material is not compromised. 

Outside the LVR, G' trend begins to decrease, while G'' values remains almost constant, indicating that the viscosity of the material increases with respect to its elasticity. At the crossover point (also called *critical stress*) the viscosity drops drastically because the material collapses and gel-sol transition occurs. 

After four days of settling, G' and G'' regained the same values of the native hydrogel, evidencing the thixotropic nature [16,26] of AC1 hydrogel and the recovery of its original morphology (data not shown).

**Figure 3:**
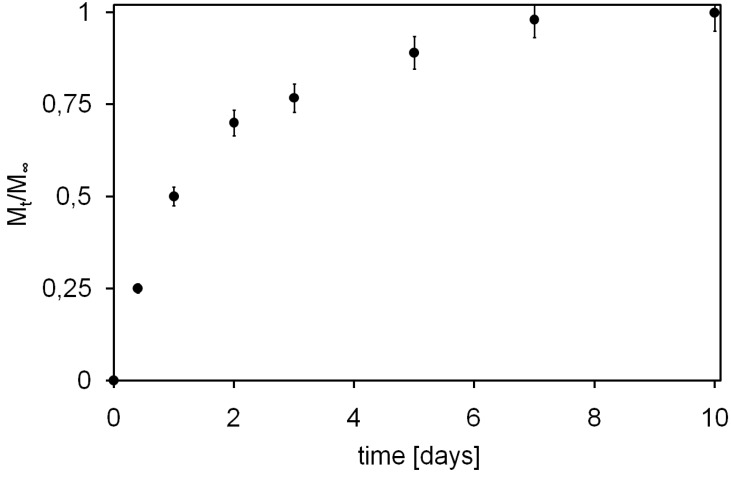
Texas-Red dextran (Tx) release profile from *AC1* (*M*_t_) expressed as unitary fraction with respect to total loaded mass (*M*_∞_).

As hydrogel injectability was tested confirming previous results [14], its ability to release drugs with high hydrodynamic radius, in accordance with SCI medical needs, was investigated: achieved results are presented in Figure 3, depicted as cumulative mass fraction of Tx released in the outer solution (M_t_/M_∞_).

Figure 3 shows a rapid initial release of Tx due to the initial high concentration gradient and to the so-called burst effect [27]. This burst is likely caused by molecules placed near solvent-hydrogel interface that can rapidly escape into the supernatant solution, as well as by molecules that can find a fast path through large pores of the hydrogel, with respect of those that have to diffuse through smaller ones, thus partially suffering a constrained molecular motion. After this initial burst, the release becomes slower and reaches an almost steady state condition after about 7 days (*plateau*). Moreover, it has to be noted that all loaded Tx was released, underlining not only the absence of any stable bonds between hydrogel polymeric network and loaded Tx but also hydrogel capacity to deliver even those molecules deeply entrapped in its core.

**Figure 4:**
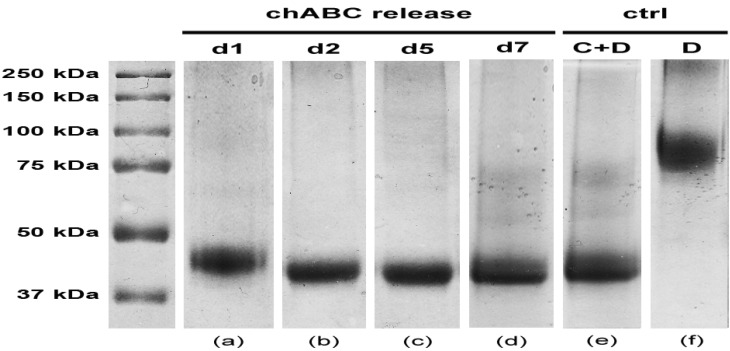
SDS-PAGE assay of enzymatic activity of chABC. Patterns of decorin degradation due to released chABC: lane **a**, release at day 1; lane **b**, release at day 2; lane **c**, release at day 5; lane **d**, release at day 7. Ctrl: lane **e**, preincubated chABC + decorin (C+D); lane **f**, pure decorin (D).

Nevertheless, besides being able to transport molecules with high hydrodynamic radius, a delivering hydrogel must preserve drug biochemical properties, *i.e.*, in this specific case chABC enzymatic activity. SDS-PAGE plot reported in Figure 4 shows, at different time points, decorin digestion products due to the chABC released from the hydrogel.

As can be noticed, each time point (1, 2, 3 and 7 days) is described by a lane (a–d) with an evident band in the range of 37–50 kDa. Specifically, this band is close to 45 kDa, which represents the molecular weight of decorin main digestion product (lane e). Indeed, decorin is known to consist of two or three asparagine-bound oligosaccharides, weighting about 45 kDa each, and a glycosaminoglycan chain. After chABC digestion just its 45 kDa core is still preserved [28,29]. These data thus confirm the maintained enzymatic activity of chABC up to 7 days, which is the expected time frame for the complete chABC delivery from the gel. 

Hence, the absolute neutrality of agarose-carbomer hydrogel was proven with respect to chABC biochemical features for the whole length of the delivery interval.

## 4. Conclusions

Up-to-date spinal cord repair strategies cannot be considered separately from degrading the glial scar, which is the main obstacle to axonal regrowth. chABC has been proven to successfully digest CSPGs, but it presents remarkable administration shortcomings. In this study, a promising agarose-carbomer hydrogel was investigated, demonstrating its capacity to release molecules with high hydrodynamic radius over a one-week period. The material showed the ability to entrap chABC molecules and exhibited coherent mechanical properties to be easily injected into the target tissue. 

Furthermore, hydrogel loading did not induce any stable bonding with chABC nor any chABC denaturation: an effective digestion of scar-like molecules, such as decorin, was observed throughout the whole expected delivery timeframe. Agar-carbomer hydrogel was thus confirmed to be a potential delivery tool for chABC in spinal cord injury repair strategies.
